# Exploring Insulin Production Following Alveolar Islet Transplantation (AIT)

**DOI:** 10.3390/ijms221910185

**Published:** 2021-09-22

**Authors:** Hien Lau, Tanja Khosrawipour, Shiri Li, Michael Alexander, Piotr Frelkiewicz, Maya Karine Labbé, Sven Stieglitz, Jonathan Robert Todd Lakey, Wojciech Kielan, Veria Khosrawipour

**Affiliations:** 1 Department of Surgery, University of California, Irvine (UCI), Orange, CA 92868, USA; michaela@hs.uci.edu (M.A.); jlakey@hs.uci.edu (J.R.T.L.); veriakhosrawipour@yahoo.de (V.K.); 2 Department of Surgery (A), University-Hospital Düsseldorf, Heinrich-Heine University, Moorenstrasse 5, D-40225 Duesseldorf, Germany; 3 Department of Surgery, Weill Medical College of Cornell University, New York, NY 10065, USA; shl4018@med.cornell.edu; 4 Center for Experimental Diagnostics and Biomedical Innovations, Wroclaw University of Environmental and Life Sciences, 50-375 Wroclaw, Poland; piotr.frelkiewicz@upwr.edu.pl; 5 School of Dentistry, Wroclaw Medical University, 50-367 Wroclaw, Poland; maya_labbe@hotmail.fr; 6 Department Pulmonary Medicine, Petrus-Hospital Wuppertal, University of Witten-Herdecke, D-42283 Wuppertal, Germany; sven.stieglitz@cellitinnen.de; 7 2nd Department of General Surgery and Surgical Oncology, Wroclaw Medical University, 50-556 Wroclaw, Poland; wojciech-kielan@wp.pl

**Keywords:** islet, insulin, aerosol, lung, drug delivery, transplantation, xenograft

## Abstract

Recent studies have demonstrated the feasibility of islet implantation into the alveoli. However, until today, there are no data on islet behavior and morphology at their transplant site. This study is the first to investigate islet distribution as well insulin production at the implant site. Using an ex vivo postmortem swine model, porcine pancreatic islets were isolated and aerosolized into the lung using an endoscopic spray-catheter. Lung tissue was explanted and bronchial airways were surgically isolated and connected to a perfusor. Correct implantation was confirmed via histology. The purpose of using this new lung perfusion model was to measure static as well as dynamic insulin excretions following glucose stimulation. Alveolar islet implantation was confirmed after aerosolization. Over 82% of islets were correctly implanted into the intra-alveolar space. The medium contact area to the alveolar surface was estimated at 60 +/− 3% of the total islet surface. The new constructed lung perfusion model was technically feasible. Following static glucose stimulation, insulin secretion was detected, and dynamic glucose stimulation revealed a biphasic insulin secretion capacity during perfusion. Our data indicate that islets secrete insulin following implantation into the alveoli and display an adapted response to dynamic changes in glucose. These preliminary results are encouraging and mark a first step toward endoscopically assisted islet implantation in the lung.

## 1. Introduction

Type I diabetes mellitus (T1DM) is an autoimmune disorder characterized by the destruction of β-cells of pancreatic Langerhans islets. A variety of secondary diseases can emerge as a result of disrupted insulin secretions and imbalanced glycemic control. For affected patients, human pancreatic islet allografts are a potential curative treatment [1,2]. The limited availability of quality human islets has encouraged attempts to perform islet xenotransplantation [3,4], which requires the use of non-human xenogeneic pancreatic islets donors. With respect to continuous experimental and clinical research on this topic for the past three decades, islet xenotransplantations have delivered promising results in both animal studies and clinical trials [5,6,7,8]. The scientific community has focused on tackling a wide range of challenges when attempting xenograft transplantations in a new host. Some of these challenges include possible interactions with the host’s immune system, endurance and longevity of the graft at its implantation site, vascular supply, and complications during and following transplantations. Additionally, the risk of disease transfer from the graft must be considered, including porcine endogenous retrovirus in the case of swine grafts [9]. This becomes an even greater issue with higher levels of immune suppression.

Moreover, with a wide range of potential implantation sites, it is quite a challenge to choose an optimal location with respect to the aforementioned challenges. As demonstrated in a recent study, islets can potentially be implanted within the alveolar space by means of aerosolization [10]. In recent years, the scientific community has expressed increased interest in aerosol-based and catheter-guided medical and pharmacological applications [11,12,13,14]. Recent studies have demonstrated the capability of aerosol-based applications to carry and deliver complex pharmaceutical particles [15,16,17]. The implantation of islets into the alveolar space can potentially solve some of the challenges clinicians and researchers currently face. One of the most important challenges is hypoxia at the transplant site. In fact, hypoxia has been identified as the most critical factor for survival and function of transplanted islets [18,19,20]. Thus, achieving optimal oxygenation of the transplanted islets is a key factor when developing new transplantation devices harvesting islets or when directly implanting islets at a chosen location [21,22]. Alveolar islet transplantation (AIT) is a new promising concept with the potential to overcome the problem of oxygen deprivation and distance to the vascular network while providing islets with nutritional supply. Until today, specific technical and applicational aspects for the transplantation have barely been studied [10]. By means of a swine lung model, this study aims to analyze islet cell distribution and as well as the extent of insulin production at the transplantation site following AIT.

## 2. Results

The application of the islets into the lung by means of an endoscopically guided catheter was possible. Both during preparation as well as while connecting the syringe containing the islet cell suspension to the MC, a tendency for islet sedimentation within the syringe was observed. Hence, the injection of the suspension had to be swift to prevent increased cell sedimentation within the syringe. No congestion of the MC nozzle was observed during the injection phase. After aerosolization was completed, thoracotomy was performed and ICG-marked islet-graft bearing lung tissue was explanted for further analyzes.

### 2.1. Islet Size Distribution and Morphology Following Aerosolization

**Figure 1:**
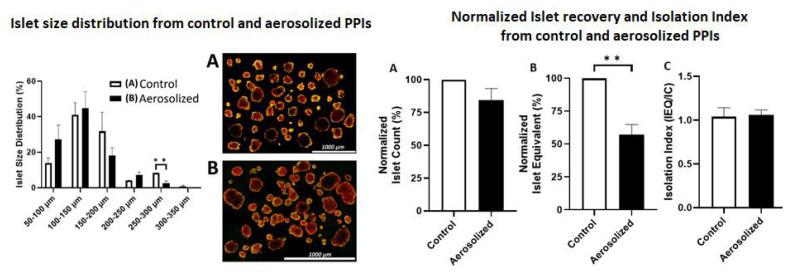
Size distribution and islet cell morphology before and after aerosolization. **Left**: Percentual size distribution (diameter in micrometer) of islets before (white column) and after aerosolization (black column). **Right**: light microscopy of islets before (**A**) and after (**B**) aerosolization. ** = *p* < 0.01.

The size of islets used was grouped into five different cohorts. The percent distribution ranged from 50–100 μm, 100–150 μm, 150–200 μm, 200–250 μm, and 300–350 μm. The total islets within these cohorts were initially (control) 14 ± 3%, 41 ± 7%, 32 ± 11%, 4 ± 0.1%, and 0.7 ± 0.7%. After aerosolization, islet size distribution changed as follows: 27 ± 8%, 45 ± 9%, 18 ± 4%, 7 ± 2%, and 0.00 ± 0.00%, respectively (Figure 1). The control group (8.33 ± 0.12%) had a significantly higher amount of large islets (250–300 μm) compared to aerosolized islets (*p* < 0.01) and a considerably lower amount of small islets (50–100 μm) (*p* < 0.05). The total diameter of islets changed in the described manner, and minimal changes in their round shape were observed. Larger aerosolized islets tended to have a more oval shape after aerosolization than islets in the control group. Islet cell equivalence was significantly lower after aerosolization while the total islet cell count was only slightly reduced. This further indicates that some of the islets were fragmented into smaller particles.

### 2.2. Placement of Aerosolized Islets in the Lungs and Structural Alteration following Transplantation

**Figure 2:**
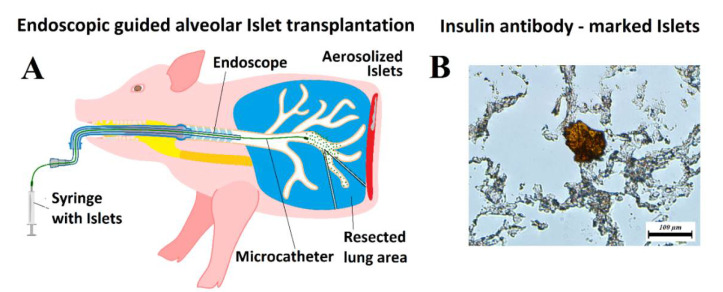
Islet transplantation. (**A**) Model of endoscopic guided alveolar islet transplantation (AIT) in a swine model. (**B**) Histology of lung tissue after AIT with verification of islet cell implantation using anti-insulin antibody-staining (C27C9, Rabbit mAB).

**Figure 3:**
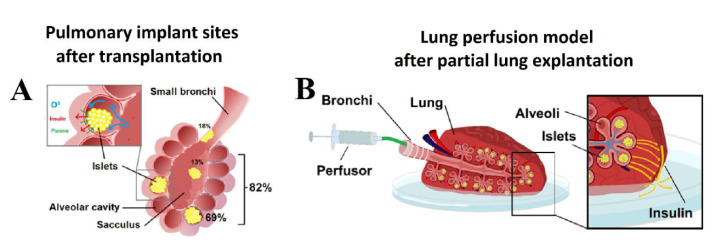
(**A**) Pulmonary transplant sites after AIT. Spatial distribution of islets at different locations in percent of total implanted Islets. (**B**) Pulmonary perfusion model for the detection of insulin production after transplantation. Continuous or pulsatile glucose stimulation can be achieved via alveolar exposure to glucose. The carrier volume (saline) of the glucose transports insulin through the lung tissue passing the visceral pleura where its concentration can be detected.

**Figure 4:**
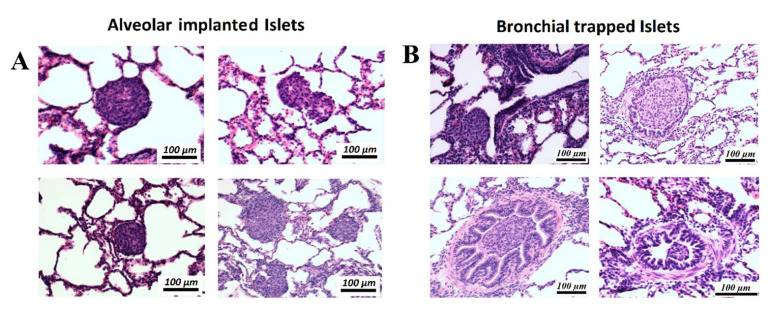
Histology (hematoxylin and eosin, H&E-staining) of implanted islets in different regions within the lung cavity. (**A**) Islets are either located in the alveolar cavity or at the center of a sacculus where multiple alveolar structures merge. **(B**) Islets trapped in different locations within the small bronchial system with partial or complete obstruction of the bronchial airway.

After aerosolization, several areas were collected for further histological analyzes by using the ICG signal as an index. In the histologic analysis, islets were clearly identified within the lung tissue (Figure 2B). Islets were detected at two major sites within the lung tissue (Figure 3A and Figure 4A,B). In the analyzed sections, a total of 84 islets were detected. Most islets (*n* = 69, 82.2%) were in the intra-alveolar space (Figure 3A). The intra-alveolar site includes both the alveolus cavity as well as the alveolar sac (sacculus). A smaller number of islets (*n* = 15, 17.8%) was detected in the small bronchiole. No conglomeration of islets was observed at any location. At the bronchial site, we observed a partial structural collapse of six islets. This amount corresponds to 40% of all islets at this location. This finding contrasted with the alveolar site, where only two islets (3%) showed disintegration and collapse. In some cases, implantation at the small bronchiole site resulted in either partial or total obstruction of that bronchiole. This partial or total obstruction was observed in eight islets (53.3%). Most islets (82.2%) were detected in the alveolar cavity as well as the alveolar sacculus. In most of these implantations, islets were at the alveolar site (69.1%). Islets at that location were in close contact with the alveolar wall. The total contact surface with the alveolar wall at the alveolar site was estimated at 60 ± 3% of the total islet diameter. A minority of islets implemented in the alveolar sacs (13.1%) resulted in loose contact with the surrounding tissue wall. 

### 2.3. Static and Dynamic Glucose Stimulation after Transplantation into the Lungs

**Figure 5:**
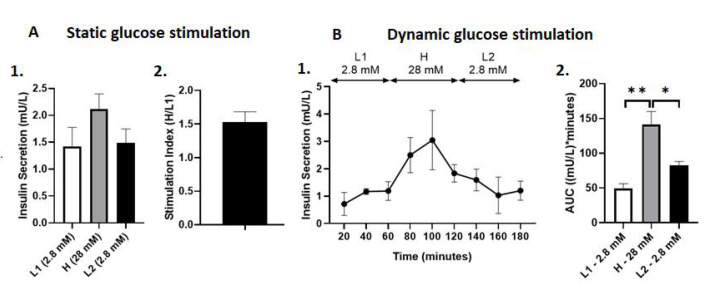
(**A**) Static glucose stimulated insulin secretion of PPIs after aerosolization into the lungs. (1) Insulin secretion (mU/L) in glucose media at varying concentrations. (2) Stimulation Index, calculated as the insulin secretion in H media over L1 media. Data are expressed as mean +/− SEM. (**B**) Dynamic glucose stimulated insulin secretion of PPIs after aerosolization into the lungs. (1) Dynamic insulin secretion (mU/L) throughout 180 min of perfusion assay. (2) Total insulin secretion expressed as AUC. * *p* < 0.05, ** *p* < 0.01. Data are expressed as mean +/− SEM.

On average, transplanted islets secreted 1.42 ± 0.36 mU insulin/L in L1 glucose media in the static glucose stimulation test. The amount of insulin secreted increased to 2.12 ± 0.28 mU insulin/L in H glucose media and decreased to 1.48 ± 0.27 mU insulin/L in L2 glucose media. However, this increase in the amount of insulin secreted in H glucose media was not significantly different when compared to either L1 or L2 glucose media (*p* = NS, Figure 3A). The stimulation index of transplanted islets was 1.53 ± 0.16 (Figure 5A). In the dynamic glucose stimulation test, the transplanted islets in the lungs showed a biphasic glucose stimulated insulin secretion capacity during perfusion (Figure 4A). The total insulin secreted by islets transplanted into the lungs expressed as AUC was significantly higher in H, with 28 mM glucose solution (141.20 ± 18.84 (mU insulin/L) × minutes), compared to the first L1 with 2.8 mM (49.58 ± 6.74 (mU insulin/L) × minutes) and second L2 with 2.8 mM glucose solution (82.86 ± 5.29 (mU insulin/L) × minutes) (*p* < 0.01, *p* < 0.05, respectively, Figure 5B). 

## 3. Discussion

### 3.1. The Delivery of Pharmacological Substances into the Lung Using Aerosolization

The delivery of pharmacological substances into the lung by means of aerosolization is an established concept in pulmonary medicine. Besides topical applications and oral intake, both aerosolization and inhalation represent two of the least invasive options of drug delivery. Therefore, multiple trials have been conducted with the aim to establish the concept of insulin uptake using lung inhalation and absorption as a viable alternative to current methods of insulin delivery [23,24]. However, significant challenges, including fluctuation in insulin uptake, have been observed following each inhalation. The extent of drug absorption depends on many factors, including composition of mucosal tissue and individual patients’ characteristics. The complexity of insulin glucose pathophysiology, including approximating the inhaled dose, mechanisms of absorption, and insulin metabolism within the lung, has impaired efforts to establish inhaled insulin as a real alternative for patients [25,26]. Following a series of studies, the FDA approved Exubera, a product designed for intrapulmonary insulin delivery by Pfizer. However, Pfizer was forced to withdraw the drug from the market due to a variety of associated problems, including its high cost, associated side effects, as well as errors in dosage [27]. 

### 3.2. Islet Cell Transplantation into the Lung Using Aerosolization

To the authors’ knowledge, no attempts have been made to investigate actual islet transplantation in the lung by means of aerosolization except for one study which discussed the potential of such a concept [10]. Endoscopically assisted AIT could reduce complication rates, dosing errors, as well as the need for repetitive inhalations as compared to inhaled insulin. At the same time, this concept offers minimal risk for potential complications when compared to conventional surgical transplantations. While bronchoscopy is not completely risk-free, the occurrence of severe complications is still considered rare [28,29]. Aerosolization of islets while preserving their mechanical stability has been previously demonstrated as technically feasible [10]. However, there are no data on the behavior of islets following AIT from an anatomical and physiological perspective. Our preliminary data are encouraging and not only indicate that AIT is feasible, but also confirm the possibility of insulin production, secretion, and diffusion in the alveoli. The next challenge is to find the optimal dosage of islets, which can now be estimated thanks to our data. However, further studies must address remaining questions on the endurance and longevity of transplanted islets. Furthermore, the interaction between lung tissue and islets must be further analyzed. The volume of islets required for transplantation seems rather insignificant compared to the total lung volume. However, some important questions remain unanswered at this point and must be addressed in future studies. These include estimations of the area of the lung occupied by the transplanted cells, as well as the extent of occupied lung tissue required for proper functioning of this concept, insight on how transplanted islets may affect respiratory function, and the amount of islets transplanted to ensure sufficient insulin production. While this concept may cause some unwanted interactions, many benefits could also be expected. 

We know that the alveoli contain the highest oxygen levels in the body and could support long-term endurance of the graft. Simultaneously, surface contact with adjacent alveoli is limited as we demonstrated that approximately 60% of the islet surface is in direct contact with the alveoli. Thus, the presented surface antigen is minimally exposed to passing cells from the immune system. Besides, this in-lumen location serves as a partial barrier to the immune system itself since it would require passing cells to enter the alveolar cavity to exert their effects.

## 4. Material and Methods

### 4.1. Porcine Pancreatic Islet Isolation and Culture

All animal procedures were performed in accordance with the University of California, Irvine Institutional Animal Care, and followed committee guidelines. On postnatal days 8–15, pre-weaned Yorkshire pigs were used as pancreatic islet donors as previously described [30]. In short, porcine pancreata were procured, isolated for 10 min, and stored in Hank’s balanced Salt solution (HBSS) on ice. The pancreata were subjected to less than one hour of cold ischemia and then chopped into 1 mm^3^ pieces. Using a Sigma Type V Collagenase (2.5 mg/mL, dissolved in HBBS, Sigma-Aldrich, St. Louis, MO, USA), chopped tissues were digested at 37 °C in a 100 rpm shaking water bath. To quench the digestion, HBSS supplemented with 1% porcine serum (Gibco-Thermo Fisher Scientific, Waltham, MA, USA) was used. Digested tissues were then filtered through a 500 μm metal mesh. After filtration, an islet maturation media composed of 50% Ham’s F-12 medium (Corning Inc., Midland, NC, USA), 50% medium 199 (Corning Inc., Midland, NC, USA), 10 mM HEPES (Sigma-Aldrich, St. Louis, MO, USA), 5 mM L-glutathione (Sigma-Aldrich, St. Louis, MO, USA), 0.6 mL/L ITS+3 (Sigma-Aldrich, St. Louis, MO, USA), 10 mM nicotinamide (Sigma-Aldrich, St. Louis, MO, USA), 100 μg/mL gentamicin sulfate (Corning Inc., Midland, NC, USA), 10 μM Trolox (Sigma-Aldrich, St. Louis, MO, USA), 200 U/L heparin (Sagent Pharmaceuticals, Schaumburg, IL, USA), 0.1 mM pefabloc (Santa Cruz Biotechnology, Santa Cruz, CA, USA), 2 mM L-glutamine (Alfa Aesar, Carlsbad, CA USA), 2.5 mM calcium chloride dihydrate (Fisher Scientific, Waltham, MA, USA), 1000 U/L DNase (Sigma-Aldrich, St. Louis, MO, USA), antibiotic/antimycotic solution (Corning Inc., Midland, NC, USA), and 10% porcine serum (Gibco-Thermo Fisher Scientific, Waltham, MA, USA) were used to culture islets for 7 days in T-150 untreated suspension flasks (Corning Inc., Midland, NC, USA) inside a 37 °C, 5% CO_2_ humidified incubator (Thermo Forma Series II 3120, Water Jacketed CO_2_ Incubators, Waltham, MA, USA). One day after culture, a complete media change was performed. On days 3 and 5, a half media change was performed. On day 7, porcine pancreatic islets (PPIs) were collected for further assessment. Due to limitations in organizing and scheduling, islet implantation could not be performed on the same day as islet cell explantation. Therefore, islets were temporarily placed in a maturation medium. 

### 4.2. Microcatheter (MC)

A suspension of islets, dissolved in 10 mL 0.9% saline solution with 0.5 mL indocyanine green dye (ICG, IC-Green-™, Akorn Inc., IL, Buffalo Green, USA), was filled into a sterile syringe. Then, 10 mL of each islet sample was delivered into the small opening of a microcatheter (MC, PW-205V Olympus Surgical Technologies Europe, Hamburg, Germany) with constant flow. The MC has a connecting device that consists of a pressure line that connects the shaft to a nozzle, which has a small opening. Using a 10 mL syringe and high manual pressure (1 mL/s), these pneumatic nebulizers generated a polydisperse aerosol stream out of the nozzle [31,32]. The MC device itself was introduced to an endoscope. It was possible to conduct the aerosolization procedure under visual control. The islet suspension was then aerosolized into the bronchial system using the MC with the described endoscopic support (Figure 2A).

### 4.3. Islet Recovery and Isolation Index

To determine islet cell count (IC) and equivalence (IEQ), an aliquot of 100 μL IEQ was collected for staining with 1 mL dithizone (DTZ, MP Biomedicals, Solon, OH, USA) for 5 min [30]. A standard stereo microscope (Max Erb, Santa Ynez, USA) attached to a 10× eyepiece granule was used to count the stained islets. Islet recovery was determined based on the percentage of IC and IEQ normalized to control islets without aerosolization [33] (Figure 1). Islet fragmentation was estimated based on the isolation index, which was determined as the ratio of IEQ divided by IC, as previously mentioned [34]. 

### 4.4. Islet Transplantation into the Lungs

Three Yorkshire pigs at 30 min postmortem were used for these experiments. They were first placed in a supine position, and an endoscope was inserted into the trachea. The endoscope with the MC was slowly advanced into the right bronchus and subsequently into the bronchial system (Figure 2A). A suspension of islets, which was dissolved into 10 mL 0.9% saline solution with 0.5 mL ICG dye, was aerosolized into the bronchial system. After aerosolization was completed, thoracotomy was performed and ICG-marked islet-graft bearing lung tissue was explanted. Part of the explanted lung was used to verify implantation using an insulin antibody (Cell Signaling Technology, Danvers, MA, USA) (Figure 2B). After confirming the presence of islets within the deep lung tissue, further histologic analyses were conducted using hematoxylin and eosin (H&E) staining to analyze the distribution of implanted islets within the tissue. 

### 4.5. Histological Analysis

To further investigate the implantation sites following islet cell transplantation, a part of ICG-marked lung tissue was subjected to histological analysis. The tissue samples with transplanted islets were fixed for 48 h in 10% neutral buffered formalin. Formalin-fixed samples were prepared for paraffin processing by serial dehydration in increasing concentrations of ethanol solution using a tissue processor (Leica TP1020, Leica Microsystems, Buffalo Grove, IL, USA). After preparation, tissues were embedded in paraffin wax using a tissue embedder (Leica EG 1150C, Leica Microsystems, Buffalo Grove, IL, USA). Paraffin-embedded tissue blocks were cut into 5 μm thin sections using a microtome (Leica RM 2255, Leica Microsystems, Buffalo Grove, IL, USA). The 5 μm sections were stained with hematoxylin and eosin (H&E) according to standard protocol. Slides were imaged using an inverted microscope (Nikon Ti-E Widefield microscope, Nikon Instruments Inc., Melville, NY, USA).

### 4.6. The Lung Perfusion Model

A resected lung area with islets marked with ICG was placed in a Petri dish with a thin layer of physiological saline solution. The bronchial branch of the resected area was tightly connected to a small tube attached to a perfusor. Lungs were placed in a perfusion chamber at 37 °C and 5% CO_2_. An initial volume as physiological saline solution was applied to ensure there was no leakage from any damaged bronchial branch distal to the main branch. After leaving the implanted islets to rest in the lung tissue for 60 min, a glucose solution was filled into the perfusor. Both static and dynamic insulin secretion tests were conducted. 

### 4.7. Static Glucose Stimulated Islet Insulin Secretion

A glucose-stimulated insulin release (GSIR) assay was used to determine the insulin secretion of transplanted islets [30] in the following order of glucose media: low glucose (2.8 mM; L1), high glucose (28 mM; H), second low glucose (2.8 mM; L2), and high glucose plus 3-isobutyl-1-methylxanthine (28 mM + 0.1 mM IBMS; H+). The fluid medium in the Petri dish was continuously monitored to detect insulin concentrations at any given time. A standard porcine insulin enzyme-linked immunosorbent assay (Porcine Insulin ELISA, Mercodia, Winston Salem NC, USA) was used to quantify the insulin concentration from the supernatant of the medium and then analyzed on a microplate reader (Tecan Infinite F200, Tecan, Morrisville, NC, USA). As such, dividing the amount of insulin in the high glucose media over the first low glucose media was used to calculate the stimulation index (SI). 

### 4.8. Dynamic Glucose Stimulated Islet Insulin Secretion

Dynamic perfusion assay experiments were performed based on previous studies [35,36]. A syringe pump was used to circulate glucose media at a rate of 0.2 mL/min. Islet-graft bearing lungs were stabilized with 2.8 mM glucose media for 45 min and then exposed to the following sequence of glucose media: 2.8 mM glucose media (L1—2.8 mM) for 60 min, 28 mM glucose media (H—2.8 mM) for 60 min, and 2.8 mM glucose media again (L2—2.8 mM) for 60 min. Serial perifusate samples were collected every 20 min and analyzed for insulin concentration. The area under the curve (AUC) was used to calculate the total insulin secretion from each glucose media concentration [37]. 

### 4.9. Statistical Analysis

All data are presented as mean ± standard error of mean (SEM). Statistical significance was evaluated using an unpaired two-sample Student’s *t*-test. *p*-values < 0.05 were considered statistically significant. GraphPad Prism (GraphPad Software 8.0.1, GraphPad Inc., La Jolla, CA, USA) was used to analyze all data.

## 5. Conclusions

This study is the first to provide significant data on AIT procedures. However, many questions on the behavior and efficacy of islets at the transplantation site remain unanswered, impacting the planning of any clinical trials. Further studies, as well as in vivo experiments, are required to assess the biological efficacy of this method with regards not only to endurance and longevity, but also associated complications of this novel concept.

## Data Availability

All relevant and supporting data is presented within the manuscript.

## Abbreviations

AIT	Alveolar Islet Transplantation
HBBS	Hank’s balanced Salt solution
H&E	Hematoxylin and Eosin
IC	Islet count
ICG	Indocyanine green
IEQ	Islet equivalence
MC	Microcatheter, PW-205V Olympus
PPI	Porcine pancreatic islets
